# Transcending Blood—Opportunities for Alternate Liquid Biopsies in Oncology

**DOI:** 10.3390/cancers14051309

**Published:** 2022-03-03

**Authors:** Bonnita Werner, Kristina Warton, Caroline E. Ford

**Affiliations:** Gynaecological Cancer Research Group, School of Clinical Medicine, Faculty of Medicine and Health, University of New South Wales, Sydney, NSW 2052, Australia; bonnita.werner@unsw.edu.au (B.W.); k.warton@unsw.edu.au (K.W.)

**Keywords:** cell-free DNA (cfDNA), ctDNA, solid tumours, liquid biopsy, precision medicine, biomarkers, ascites, cerebrospinal fluid, urine, pleural effusion

## Abstract

**Simple Summary:**

Cell-free DNA—DNA that has been expelled from cells and can be isolated from blood plasma and other body fluids—is a useful tool in medicine, with applications as a biomarker in diagnosis, prognosis, disease profiling, and treatment selection. In oncology, the ease of access to the tumour genome is a major advantage of cell-free DNA, but while this has led to significant research in blood, other body fluids have not received equal attention. This review article summarises the current research into cell-free DNA in non-blood body fluids, highlighting its values and limitations, and suggesting the direction of future studies. We conclude that cell-free DNA from non-blood body fluids may provide additional information to supplement traditional biopsies, allowing informative and improved patient care across many cancer types.

**Abstract:**

Cell-free DNA (cfDNA) is a useful molecular biomarker in oncology research and treatment, but while research into its properties in blood has flourished, there remains much to be discovered about cfDNA in other body fluids. The cfDNA from saliva, sputum, cerebrospinal fluid, urine, faeces, pleural effusions, and ascites has unique advantages over blood, and has potential as an alternative ‘liquid biopsy’ template. This review summarises the state of current knowledge and identifies the gaps in our understanding of non-blood liquid biopsies; where their advantages lie, where caution is needed, where they might fit clinically, and where research should focus in order to accelerate clinical implementation. An emphasis is placed on ascites and pleural effusions, being pathological fluids directly associated with cancer. We conclude that non-blood fluids are viable sources of cfDNA in situations where solid tissue biopsies are inaccessible, or only accessible from dated archived specimens. In addition, we show that due to the abundance of cfDNA in non-blood fluids, they can outperform blood in many circumstances. We demonstrate multiple instances in which DNA from various sources can provide additional information, and thus we advocate for analysing non-blood sources as a complement to blood and/or tissue. Further research into these fluids will highlight opportunities to improve patient outcomes across cancer types.

## 1. Introduction

The field of oncology has benefitted from the advancements in cell-free DNA (cfDNA) research since its discovery in the mid-20th century [1]. With the ongoing progress in molecular biology, the potential of cfDNA as a clinical tool is compelling. Though cfDNA that circulates in the blood (cirDNA) has proven clinical value—with multiple applications already in clinical practice [2]—it has substantial limitations, including low concentrations and limited release of cell-free tumour DNA (ctDNA) into the blood stream by cancers in certain anatomical locations, such as the brain. While other body fluids have not received the same research attention as blood, early studies indicate that cfDNA isolated from non-blood sources may have a unique clinical utility, especially in situations where blood and tissue specimens are inaccessible or dated [3].

**Figure 1:**
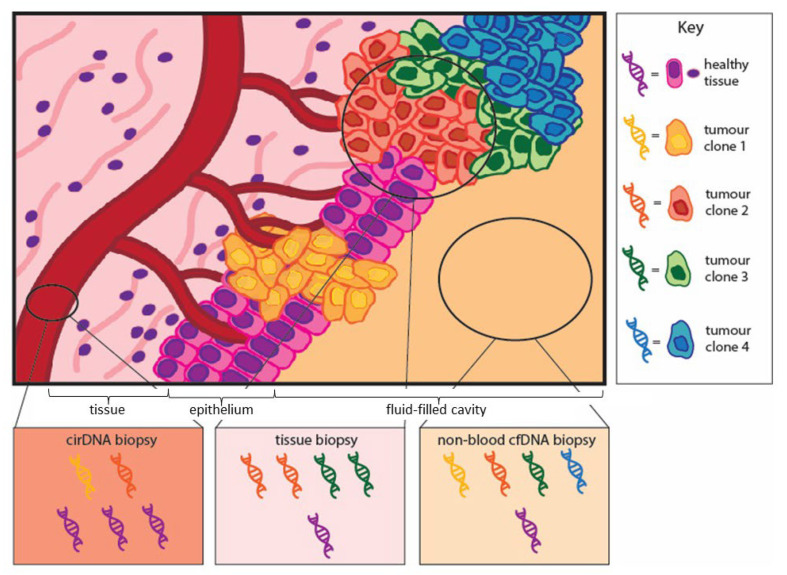
Tumour clonal representation in traditional tissue biopsies and liquid biopsies. Traditional tissue biopsies are rich in tumour DNA but can be naïve to tumour heterogeneity. Liquid biopsies of circulating cell-free DNA (cirDNA) in blood are diluted with DNA from normal tissue. Liquid biopsies of cell-free DNA from non-blood sources can capture disease heterogeneity better than tissue biopsies with far less dilution with healthy DNA than cirDNA.

Traditional tissue biopsies can provide insights to inform clinical management. However, they come with crucial limitations; tissue biopsies are site-restricted, invasive and can misrepresent the heterogeneity of a cancer [4]. They can also yield insufficient material for genetic analysis in up to 25% of cases [5]. The discovery that body fluids contain disease-related constituents gave rise to the concept of an informative and minimally invasive ‘liquid biopsy’. Sampling fluid allows a non-biased, random selection of tumour-derived factors, capturing tumour heterogeneity in a way that cannot be achieved from a single tissue biopsy (Figure 1) [6]. This has obvious benefits in oncology and has been investigated, and in some cases implemented, for cancer diagnosis, patient monitoring, and tumour molecular analysis in a range of cancer types [7,8].

cirDNA has been of interest as an analyte in liquid biopsies since 1977, when it was first found to contain ctDNA [9]. In healthy individuals, cirDNA is present at low levels (10–15 ng/mL blood), though it does increase during tissue stress instigated by exercise, inflammation, or injury [10]. In cancer patients it can be up to 10-fold higher, especially with metastatic disease, partially due to ctDNA contribution [9]. Multiple commercial cirDNA-based oncology assays have been approved for early detection (for example, Epi proColon^®^ for colorectal cancer) [11], disease monitoring (for example, COLVERA™ for monitoring the recurrence of colorectal cancer) [12], and precision medicine (for example, Cobas^®^ EGFR Mutation Test v2, for the prediction of non-small cell lung cancer responses to epidermal growth factor receptor (EGFR) inhibitors) [13,14].

**Figure 2:**
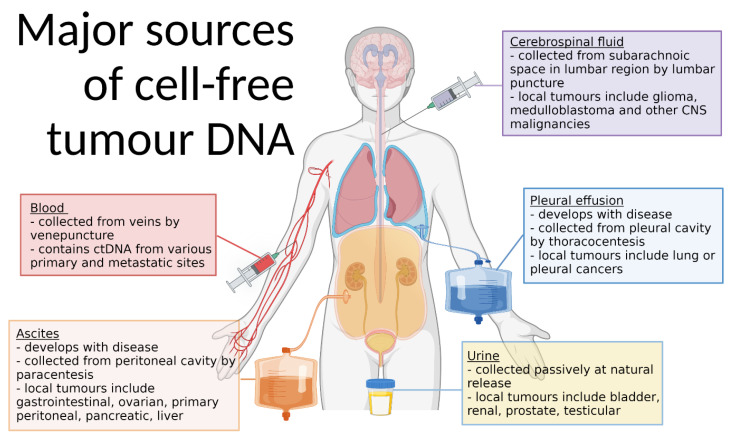
Major sources of cell-free tumour DNA. ctDNA can be sourced from many body fluids, including blood, ascites, cerebrospinal fluid, pleural effusions, and urine. These fluids, though often also containing ctDNA from non-local tumours, are particularly advantageous due to their contact with tumours in the anatomical locations indicated. All fluids are collected by minimally invasive or non-invasive methods, and pleural effusions, ascites, and sometimes cerebrospinal fluid are incidentally collected as symptom management. Created with BioRender.com (accessed on 8 February 2022). CNS, central nervous system.

Although other body fluids can be arguably more accessible and less invasive to collect than blood, they have not been afforded equal attention, with ctDNA not discovered in urine until 1995 [15]. ctDNA has since been found in saliva [16], sputum [17], semen [18], faeces [19], and cerebrospinal fluid (CSF) [20]. With respective proximity to cancers of the bladder, head and neck, lungs, prostate and testes, gastro-intestinal tract, and central nervous system (CNS), these fluids may have unique advantages (Figure 2) [21]. Additionally, pathological fluids, such as pleural effusions and ascites (which arise only in a diseased state), are known ctDNA sources [22,23]. These fluids are drained therapeutically and may present more direct insight into their associated cancers than blood; however, they too have received limited research attention [24].

Several advantages of non-blood sourced cfDNA have been described in the literature, including the tendency to be a richer and more diverse source of ctDNA, carrying additional information not detected in samples from blood or tissue [19,20,25]. Though promising, further research is needed to establish the full scope of the applications and limitations of non-blood sourced cfDNA. In this review, we evaluate the evidence for non-blood cfDNA as a liquid biopsy substrate and identify areas for future research to focus on in order to facilitate progress towards clinical implementation.

## 2. Reliable Molecular Profiling of Disease

### 2.1. Does Non-Blood Sourced cfDNA Recapitulate Tumour Tissue Profile?

The most critical requirement for liquid biopsies is a demonstrated ability to recapitulate information from the solid tumour, and there is evidence that non-blood-sourced cfDNA can do this very effectively. Table 1 condenses the findings of sequencing reports comparing cfDNA from various body fluids versus tissue. We indicate the percentage of cases in which any additional mutations were discovered in a tissue or fluid sample within an individual (or, where indicated, within total mutations across a cohort), and cases where there was concordance between the two sources. A report from 2021 examined the concordance between the cfDNA from non-blood fluids and matched tissue biopsies, in variant alleles and their frequencies [26]. Pleural fluid, peritoneal fluid, pericardial fluid, and fine needle aspirate was collected for diagnostic cytology from 23 people with various cancer types, and the results were compared to formalin-fixed paraffin-embedded (FFPE) tissues [26]. The average tumour proportion, estimated by variant allele frequency (VAF), was similar between the two DNA sources (24% in fluid compared to 26% in cell blocks) [26]. Ninety percent of the variants detected in cell blocks were concordant with the fluid samples, and identical variant profiles were identified in 74% of sample pairs [26]. Discordant variants may represent the less dominant clones; where reported, VAF was lower than average in discordant values (3% and 8%) [26]. Another study, also comparing multiple serous fluids, similarly found concordance between matched tumour and liquid biopsies, with additional information from the latter in 30% of cases [27]. These studies were not able to discern an advantage of certain fluids or tumour locations over others, due to low cohort numbers.

Studies that focus on individual fluids support the additional information present, with cfDNA shown to harbour unique mutations in CSF [28], urine [29], ascites, and pleural effusions [22]. In a cohort of patients with glioblastoma (GBM), all GBM-related coding mutations detected in tumour tissue were similarly detected in CSF, but additional GBM-related changes were seen in the CSF cfDNA of 5/9 patients [28]. In two case studies of recurrent medulloblastoma, where driver mutations were found to differ between the initial and recurrent disease, cfDNA from CSF was found to accurately detect the changes [30]. Similarly, cfDNA from pleural effusions has been found to correspond to matched tumour tissue [31,32]. Among 29 patients with lung cancer, 93% of the driver mutations identified in tumour tissue were similarly detected in the cfDNA from pleural effusions [33]. In ascites fluid from a cohort of mixed KRAS-positive and wild-type tumours, KRAS mutations were detected in 100% of the KRAS-positive cohort, and in an additional 19% of tumours deemed wild-type by tissue biopsy [34]. Sputum and saliva, however, though similarly representative of tumour tissue, are less sensitive sources for cfDNA analysis [17,35].

The heightened mutation detection in non-blood fluids may indicate their capacity to capture intra-tumour heterogeneity. However, tumour heterogeneity may not be the only source of the additional mutations in cfDNA. Alternative explanations include a higher vulnerability to artefactual DNA changes in the extracellular space, technical errors in sequencing caused by the unique characteristics of cfDNA, or the inheritance of unfavourable mutations preceding cell death. These avenues should be explored to reach confidence in cfDNA profiling in the clinical setting.

**Table 1:** Concordance in variants detected in tumour tissue and cfDNA from body fluids.

Fluid	Concordance between Sources	Additional Variants in Tissue	Additional Variants in Fluid	Source
Ascites	80% (*n* = 5)	20% (*n* = 1)	0% (*n* = 0)	Perrone, M.E. et al., 2021 [26]
	100% (*n* = 2)	0% (*n* = 0)	0% (*n* = 0)	Yang, S.R. et al., 2019 [27]
	10% (*n* = 1)	60% (*n* = 6)	70% (*n* = 7)	Han, M.R. et al., 2020 [25]
	88% (*n* = 23)	0% (*n* = 0)	12% (*n* = 3)	Leick, K.M. et al., 2020 [34]
CSF	44% (*n* = 4)	0% (*n* = 0)	56% (*n* = 5)	Duan, H. et al., 2020 [28]
Pleural effusions	75% (*n* = 12)	19% (*n* = 3)	6% (*n* = 1)	Perrone, M.E. et al., 2021 [26]
	75% (*n* = 6)	0% (*n* = 0)	25% (*n* = 2)	Yang, S.R. et al., 2019 [27]
	100% (*n* = 6)	0% (*n* = 0)	0% (*n* = 0)	Mokanszki, A. et al., 2021 [32]
	58% (mutations = 96)	18% (mutations = 29)	24% (mutations = 40)	Tong, L. et al., 2019 [33]
	50% (*n* = 7)	36% (*n* = 5)	21% (*n* = 3)	Guo, Z. et al., 2019 [36]

*n*—number of people, CSF—cerebrospinal fluid.

### 2.2. What Are the Distinctive Features of Non-Blood Sourced Cell-Free DNA Compared to cirDNA?

#### 2.2.1. Is There an Explanation for the Different ctDNA Concentrations in Blood and Non-Blood Fluids?

One of the major limitations of working with cirDNA is the high ctDNA dilution with white blood cell cfDNA, which may contain sequence variants arising from clonal haematopoiesis. For example, in an investigation that used high intensity sequencing to compare somatic mutations in cfDNA, white blood cells, and tumour biopsies, over 50% of the mutations detected in cirDNA from cancer patients were due to normal clonal haematopoiesis [37]. A key advantage of cfDNA in non-blood body fluids is the characteristically high ctDNA fraction, which makes tumour DNA easier to sequence and detect.

Improved ctDNA detection rates have been observed in cfDNA from non-blood sources compared to blood [29], with the exception of saliva [35] and sputum [17,38]. This has been repeatedly demonstrated in pleural effusions secondary to lung cancer, with an increased sensitivity to EGFR mutations in malignant pleural effusions over plasma, as well as over cell pellets from the effusions [31,32,33,36]. Furthermore, in two independent cohorts of lung adenocarcinoma patients, higher ctDNA abundance was seen in other body fluids, including ascites, pericardial effusions, and CSF, compared to blood plasma [39,40]. The clinical implications of this are clear and have been demonstrated in a multi-cancer study of precision medicine; 36% of participants, despite having no detectable driver mutations in blood, possessed driver mutations detected in the cfDNA from serous body fluids—which had a higher total cfDNA concentration and a higher VAF—allowing the guidance of personalised treatment [41].

An explanation for the high ctDNA abundance may be the proximity of these fluids to the tumour. Ascites—common in cancers of the abdomen and pelvis, including ovarian, gastro-intestinal, pancreatic, and uterine cancers [42,43]—is generally in immediate proximity to tumours, and often contains suspended cancer cells. In fact, in ovarian cancers, suspended cancer cells are the predominant mode of metastasis, which is mainly localised to the peritoneum [44]. The encapsulation of disease in the peritoneum may limit ctDNA access to the circulation [45]; in a cohort of patients with confirmed KRAS-positive pancreatic ductal adenocarcinoma, KRAS mutations were detected in the cirDNA of 18 of 19 patients with metastases outside of the peritoneum (for example in the lungs or liver), but in only 5 of 26 patients with disease confined to the peritoneum [46]; in another study of patients with peritoneal carcinomatosis, next-generation sequencing of cirDNA detected tumour fractions in the cirDNA of only 31 of 80 patients (the presence or absence of extraperitoneal lesions in this cohort was not reported) [47]. In an investigation of colorectal cancer, KRAS/BRAF mutations were detected in cirDNA by digital droplet PCR in only 20% of mutation-carrying patients with peritoneal confined disease, compared to 93% of patients with liver metastases, but when ascites was similarly tested, mutations were detected in 100% of cases (*n* = 20) [48].

Other contexts in which fluid proximity appears to be important in ctDNA dissemination and detection include CSF in cancers of the CNS [49] and urine in renal cell carcinoma [50]. cirDNA dissemination is hypothesised to be restricted by the blood-brain barrier, hampering the access of CNS-derived ctDNA into the bloodstream [51]. In a 2014 analysis of 640 cancer patients with various primary tumours, ctDNA detection in cirDNA was significantly lower in patients with medulloblastomas and gliomas, compared to patients with local or advanced carcinomas external to the CNS (40% and 10%, respectively, compared to 50% to >75%, depending on disease stage) [52]. Where detected, the ctDNA concentration was lower in patients with primary CNS tumours [52]. This was backed by a more recent report in which ctDNA was detected in only 1 of 13 medulloblastoma patients’ plasma samples, and 10 out of 13 CSF samples [30]. Another group similarly found ctDNA to be present in the CSF of almost 75% of patients with primary CNS cancer [49]. In renal cell carcinoma, where there is similar proximity to both urine and blood, increased detection of ctDNA has been found in urine compared to plasma, [50]. In contrast, the detection of ctDNA was lower in urine than in blood in lung cancer patients, where the urine proximity to the tumour site is clearly less [38].

An alternative explanation for the high ctDNA concentrations in pleural effusions and ascites may be the presence of different clearance pathways compared to cirDNA. In healthy individuals, low levels of cirDNA can be attributed to efficient clearance via the liver, by phagocytosis, or by enzymatic cleavage [53,54,55,56]. In conditions where cirDNA levels are elevated, such as cancer, clearance is less efficient and cirDNA accumulates; however, the half-life remains less than two hours [53]. As the filtration mechanism of other body fluids differs to blood, clearance may be exceeded by accumulation, explaining the high concentration.

cfDNA clearance from ascites and pleural effusion may be negatively impacted by compromised lymphatic drainage. In healthy individuals, there is an equilibrium in fluid movement into and out of the peritoneal cavity, with the total volume settling at approximately 50 mL [57]. However, with malignant ascites and pleural effusion, increased fluid production and leaky blood vessels, largely due to VEGF upregulation, pair with a decreased lymphatic drainage, due to the ectopic cell obstruction of lymphatic stomata, resulting in fluid accumulation [24,58,59]. A similar process is observed in pleural effusions [60,61]. As jeopardised lymphatic drainage is a prerequisite feature of these pathological fluids, the passage of cfDNA to the liver for clearance may be reduced [58].

Since a major source of cfDNA in circulation is apoptotic and necrotic cells, the prolonged half-life in non-blood body fluids holds clinical implications [62]. The cfDNA pool may over-represent DNA from clonal sources that are no longer prevalent, and the cfDNA may have significant exposure to elements causing molecular damage. Thus, it is possible that cfDNA from non-blood body fluids contains irrelevant or artefactual DNA changes.

The DNA half-life in these body fluids should be investigated. cfDNA in saliva, in which low total ctDNA concentration has been reported, has a half-life of 3 h; however, cfDNA in urine, in which a higher ctDNA concentration has been reported, has a half-life too short to be reliably measured [63]. It is therefore difficult to estimate the effect of cfDNA degradation on ctDNA abundance. To the best of our knowledge, no studies have investigated the half-life of cfDNA in ascites fluid or pleural effusions. Further research is required to delineate the mechanism and effect of cfDNA clearance in non-blood body fluids.

#### 2.2.2. Is There Concordance between ctDNA in Blood and Non-Blood Fluids?

cirDNA is utilised clinically in various contexts, though ctDNA from blood can have a low concordance with the primary tumour in mutation profile [64,65]. Additional DNA changes have been detected in the ctDNA from body fluids compared to blood, indicating that cfDNA from non-blood sources may provide information to supplement traditional or blood-based biopsies.

cfDNA from non-blood sources often holds more tumour-derived mutations than cirDNA (Table 2) [41,66,67]. This is inconsistent with one report, where NRAS mutations were detected in an additional 25% of the cohort’s cirDNA compared to pleural effusion cfDNA (*n* = 8); however, this cohort was of patients with blood cancers, with findings therefore supportive of our previous hypothesis on proximity to the tumour [68].

The epigenetic profiles in serous fluids are less widely researched than mutations. However, evidence points towards conserved methylation patterns in ctDNA from non-blood fluids, similar to blood, with a report of a lung cancer-specific methylation panel designed for blood cfDNA also distinguishing benign from malignant pleural effusion and ascites (though matched bloods were not compared for concordance) [69]. The methylation of cfDNA in urine has also been investigated for its diagnostic potential for renal cell carcinoma [70].

The most comprehensive liquid biopsy information will likely be obtained from the complementary analysis of cirDNA, as well as non-blood sourced cfDNA. Han and colleagues collected solid tumour and ascites samples from the debulking surgery of 10 epithelial ovarian cancer patients, along with blood samples from within one week prior to surgery [25]. Compared to the number of mutations found in the cfDNA from ascites, the tumour tissue DNA captured just 32% of total mutations and the cirDNA captured 23% [25]. However, unique mutations were found in ascites cfDNA in 70% of cases, but also in solid tissue in 70% of cases, and in cirDNA in 40% of cases, indicating the value of analysing DNA from multiple sources [25].

**Table 2:** Concordance in variants detected in cfDNA from blood plasma and body fluids.

Fluid	Concordance between Sources	Additional Variants in cirDNA (Blood)	Additional Variants in cfDNA (Non-Blood)	Source
Ascites	100% (*n* = 7)	0% (*n* = 0)	0% (*n* = 0)	Villatoro, S. et al., 2019 [41]
	86% (mutations = 6)	0% (mutations = 0)	14% (mutations = 1)	Zhou, S. et al., 2018 [71]
	10% (*n* = 1)	40% (*n* = 4)	80% (*n* = 8)	Han, M.R. et al., 2020 [25]
CSF	25% (*n* = 4)	0% (*n* = 0)	75% (*n* = 8)	Villatoro, S. et al., 2019 [41]
	53% (*n* = 10)	0% (*n* = 0)	47% (*n* = 9)	Suryavanshi, M. et al., 2020 [67]
	15% (*n* = 2)	8% (*n* = 1)	77% (*n* = 10)	Escudero, L. et al., 2020 [30]
Pleural effusions	75% (*n* = 6)	25% (*n* = 2)	0% (*n* = 0)	Ozeki, M. et al., 2019 [68]
	100% (*n* = 4)	0% (*n* = 0)	0% (*n* = 0)	Villatoro, S. et al., 2019 [41]
	29% (*n* = 14)	36% (*n* = 5)	43% (*n* = 6)	Guo, Z. et al., 2019 [36]
	100% (*n* = 6)	0% (*n* = 0)	0% (*n* = 0)	Zhou, S. et al., 2018 [71]

*n*—number of people, CSF—cerebrospinal fluid.

#### 2.2.3. Is There a Difference in the Biological Origin of ctDNA in Blood and Non-Blood Fluids?

The biological characteristics of cirDNA, such as its origin, have been extensively studied [53,72]. However, with far less data on non-blood cfDNA, it is unclear how transferable our knowledge of cirDNA is.

cirDNA exists as extracellular double-stranded DNA freely suspended in blood. A range of mechanisms for cirDNA release have been proposed, including apoptosis, necrosis, active release, oncosis, pyroptosis, phagocytosis, and neutrophil extracellular trap release [53,73,74]. The release process generally results in a distinctive, ladder-like fragmentation pattern that reflects cleavage between nucleosomes, with the nucleosomes protecting the DNA from further degradation [75]. The size profile of cirDNA gives an insight into its origin: cirDNA fragments of ~167 base pairs are associated with apoptosis where they arise from caspase-dependent cleavage [53]; longer cirDNA fragments may originate from necrotic cells or extracellular vesicles released actively by live cells [73,74]; tumour derived cirDNA is enriched in smaller, mono-nucleosomal fragments of 90–150 base pairs [76,77,78].

There is some debate as to whether similar conclusions based on size analysis can be drawn regarding non-blood cfDNA. cfDNA with a similar size profile to blood has been reported in various fluids [22,32,79,80], and tumour enrichment in short cfDNA fragments has been demonstrated in CSF and urine [81,82]. However, distinct size profiles have also been observed. Trans-renal cirDNA is known to cross the kidney barrier more efficiently in fragments of approximately 100 base pairs or less, perhaps naturally enriching for ctDNA [83]. cfDNA samples with fragment sizes over 1000 base pairs are more common in semen from prostate cancer patients than in healthy controls or benign prostate hyperplasia patients [18,84]. In 2021, Yu et al. identified high levels of long cfDNA fragments in malignant pleural effusions from 11 of 30 patients with non-small cell lung cancer [85]. After isolating the high molecular weight cfDNA and comparing it to the more fragmented DNA, they identified a significant increase in cancer-unrelated (likely artefactual) mutations in the high molecular weight fraction, potentially giving false estimates of the tumour mutational burden [85]. High molecular weight DNA, albeit of a different size, was observed in a similar proportion of malignant ascites from ovarian cancer patients, though sequencing was not performed [23]. Further characterisation of the size of ctDNA from non-blood fluids is necessary to clarify the technical and biological implications.

#### 2.2.4. Is Non-Blood cfDNA Similarly Affected by Protocol Technical Parameters?

Technical factors, including extraction kits, quantitation techniques, and storage methods have been extensively studied in cirDNA, with significant impacts on assay outcomes reported [86,87]. Given the physiological distinctions between blood and non-blood sourced cfDNA, it is unclear whether similar technical outcomes are maintained.

DNA extraction kits have varying efficiency in recovering cirDNA, thus poor kit selection can lead to inaccurate results and poor performance in downstream assays [86,88]. Yield variations have been shown to be largely influenced by biased fragment size selection, with a reduced recovery of short (50–100 bp), potentially tumour-enriched fragments [89]. Furthermore, the storage of plasma before cirDNA extraction can lead to a 25% decrease in cfDNA yield per year (Yuwono, N.L., et al. in press, CCLM.2021.1152.R1).

Without insight into the effect of these technical parameters on cfDNA from body fluids, the impact on cfDNA quantitation is unclear. Commercial circulating DNA extraction kit performance should be evaluated for use with different fluid types. Adapted protocols, specific to non-blood body fluids, should be developed, ideally allowing higher input volumes to account for the large volumes of ascites and pleural effusion that can be drained. Furthermore, the long-term stability and storage requirements of non-blood fluid cfDNA should be ascertained. We have shown cell-free DNA from ovarian cancer-related ascites to be stable in terms of size profile and concentration over three days at 4 °C [23]. However, this should be further explored in a long-term time-course, comparing yield over time in different storage conditions to facilitate the inclusion of these sample types in clinical trials.

This may have a translational impact, as cirDNA concentration has been linked to tumour burden: cirDNA concentration has been suggested as a biomarker of tumour burden in childhood neuroblastoma (*n* = 79) [90]; as a prognostic indicator for overall survival in metastatic melanoma (*n* = 43) [91]; and as an indicator of response to first and second-line taxanes in metastatic castration-resistant prostate cancer (*n* = 571) [92]. To the best of our knowledge, the cfDNA concentration in body fluids has not been linked to prognosis; however, cfDNA in pleural effusion has been found to be significantly more concentrated than matched cirDNA [33]. Thus, factors affecting the yield and stability of cfDNA from non-blood fluid should be further investigated, particularly for confidence in considering total cfDNA concentration as a biomarker of disease burden.

Another technical consideration is non-blood cfDNA performance in existing sequencing platforms. Several commercial next-generation sequencing methods have been optimised for use with cirDNA, accommodating a small fragment size, low total concentration, and low tumour DNA content. However, the comparison of these methods with tissue-specific protocols has revealed a low concordance within patients [64]. In one study comparing the 65 genes common to both assays, an average of 4.82 genes were altered in the tissue and 2.96 were altered in the cirDNA [65]. This may be due to the dilution of ctDNA with genomic cirDNA, decreasing the sensitivity of cirDNA-specific assays relative to tissue-specific assays; discordance between outputs was significantly more likely with VAFs of <1% [64]. In similar studies where participants had a verified level of >10% ctDNA, the concordance in somatic mutations and copy number variations was 88% and 80%, respectively [93]. The increased ctDNA concentration in non-blood body fluids may, therefore, improve performance in established sequencing platforms.

## 3. Where Are Non-Blood Liquid Biopsies Clinically Applicable?

The analysis of traditional tissue biopsies is a common clinical practice, particularly for diagnosis and disease profiling. However, the invasiveness and expense of tissue biopsies can limit the situations in which they are applicable. The major advantage of a liquid biopsy over a standard tissue biopsy is the reduction in the risks and costs associated with the latter. Furthermore, increased accessibility allows sampling when biopsies or surgery may not typically be undertaken, such as at disease progression and the acquisition of resistance [6].

cirDNA is an attractive substrate for widescale clinical testing for multiple reasons, but largely due to the ease of access to blood: blood tests are generally acceptable at routine medical appointments [94], blood sampling is convenient and can be safely done in most circumstances, and DNA changes in blood can be traced to cancers of many primary sites [95]. Though non-blood body fluid biopsies may not always share these traits, their additional perspective may provide important supplementary information, aiding in diagnosis, disease monitoring, and precision medicine.

Due to the prior mentioned benefits of blood tests, cirDNA-based liquid biopsies are particularly well placed as screening tools for early cancer detection. Passively collected non-blood based liquid biopsies, such as urine, stool, and saliva are similarly appropriate for this purpose. Population-wide colorectal cancer self-screening tests, involving personal faecal collection, demonstrate evidence of the utility of easily collected samples, having reduced colorectal cancer mortality by up to 62% [96]. However, not all body fluids are appropriate for this purpose. In the case of pleural effusion and ascites, which are indicative of advanced disease, the objectives of early diagnosis or recurrence detection are redundant, though they may facilitate diagnosis. CSF sampling, being more invasive and involved than blood sampling, is also not appropriate for early disease screening, but may aid in confirming diagnosis where suspicions are high.

Non-blood fluids may serve better in other circumstances, such as disease monitoring. A key exclusion of the utility of cirDNA in oncological testing is in cancers of the CNS, where ctDNA presence in blood is restricted, possibly due to the blood-brain barrier [51]. ctDNA from CSF has been shown to recapitulate genomic alterations in solid paediatric medulloblastoma and to correctly stratify molecular features, facilitating clinical management, and disease monitoring [30]. Tissue biopsies of CNS tumours can be particularly challenging or impossible, so a sensitive CSF liquid biopsy is a desirable alternative for diagnosing and monitoring localised CNS tumours. Furthermore, there are instances where blood draws are detrimental to the patient. Advanced cancer is frequently coupled with anaemia and there are concerns over excessive blood draws in critically ill patients [97,98]. Here, less invasive specimens, such as urine, may be a more appropriate analyte for disease monitoring [3]. As well as containing local ctDNA contributions in the case of urothelial and prostate cancers [99,100], urine has been shown to contain ctDNA derived from the circulation, representative of cancers throughout the body [101,102,103]. Ascites and pleural effusions, being therapeutically drained as part of patient care, are another avenue of access to ctDNA that avoids imposing additional procedures on unwell patients. As these fluids typically reaccumulate, they also present an opportunity for serial disease monitoring.

Non-blood fluids are also excellent templates to enable precision medicine and informed disease management, particularly when tissue is inaccessible or out-dated and blood can be outperformed. A case study involving a patient with stage IV gastric cancer, with no tumour tissue available for sequencing, compared cirDNA from plasma with cfDNA from ascites and pleural effusion as templates for personalised medicine selection [71]. A high concordance in the mutational spectra was found between sample types, but ascites contained the highest mutant allele frequencies in all common aberrations detected [71]. The findings enabled the prescription of a targeted therapy, demonstrating an instance where non-blood body fluid could provide the most clinically pertinent information in lieu of a tissue biopsy [71].

## 4. Conclusions

We have found growing evidence to support the strength of the use of cfDNA from non-blood sources as liquid biopsy templates, providing clinically actionable information across various solid cancers. Body fluids, particularly pleural effusions, ascites, CSF and urine, can provide accessible additional information to supplement or substitute for tissue biopsies and blood liquid biopsies, where they are inappropriate or insufficient. Further research into the technical concerns highlighted in this review, including potential artefacts, stability, and performance with non-specific extraction kits, will facilitate a swift transition into clinical practice, supported by the existing platforms already developed for liquid biopsies of blood. The described advantages of non-blood cfDNA, including high tumour content and comprehensive disease profiling, may lead to a more precise understanding of an individual’s disease, being of growing importance in the era of precision medicine.

## Abbreviations

cfDNA	cell-free DNA
cirDNA	circulating cell-free DNA, referring specifically to cell-free DNA in blood plasma
ctDNA	tumour-derived cell-free DNA
